# Overcoming Giant Verruca Vulgaris Treatment Obstacles With Siddha Medicine: A Case Report

**DOI:** 10.7759/cureus.73945

**Published:** 2024-11-18

**Authors:** Christian Gnanaraj Johnson, Saravanasingh Karan Chand Mohan Singh, Ramamurthy Murugan, Gayatri R, Vinayak S

**Affiliations:** 1 Department of Noi Naadal, National Institute of Siddha, Chennai, IND; 2 Department of Udal Koorugal (Anatomy), National Institute of Siddha, Chennai, IND; 3 Research, Siddha Pharmacology, Siddha Central Research Institute, Chennai, IND

**Keywords:** giant verruca vulgaris, human papillomavirus (hpv), hyperkeratotic skin lesions, pachaieruvai, siddha medicine

## Abstract

Verrucae vulgaris are infectious keratotic lesions caused by the human papillomavirus (HPV), frequently impairing an individual's quality of life, mainly when situated on the facial region. Verruca vulgaris constitutes a prevalent dermatological issue with diverse repercussions for those affected. The interaction among HPV, the immune system, and treatment methods requires a sophisticated comprehension of this illness. The need for more proficient management of this ailment constitutes a considerable challenge for the healthcare industry. Topical corticosteroids, cryo-therapeutic techniques, laser-based treatments, and immunotherapeutic modalities, including photodynamic therapy, are recognized as costly alternatives. Siddha external medicine offers a therapeutic strategy that employs suitable methodologies. This case report elucidates the clinical presentation of a 60-year-old female exhibiting hyperkeratotic, elevated, non-painful growth lesions localized to the left malar region, which have persisted for six years. She was diagnosed with giant facial verrucae vulgaris based on the observed clinical manifestations. The patient received external therapeutic intervention utilizing Siddha medicine, particularly applying *pachaieruvai*. The intervention resulted in the complete excision of the lesion, yielding aesthetically pleasing outcomes without any adverse reactions or recurrence observed during subsequent follow-up assessments.

## Introduction

Verruca vulgaris, commonly referred to as the ordinary wart, constitutes a non-cancerous epithelial neoplasm that arises as a direct consequence of an infection induced by human papillomavirus (HPV), which is known to be a prevalent viral pathogen. While these lesions typically do not present with any painful symptoms, they can lead to significant aesthetic concerns and substantial psychological distress owing to their visible characteristics [1]. The underlying mechanisms that drive the development of verruca vulgaris involve the integration of HPV genetic material into the cellular genome of the host, which subsequently triggers an abnormal proliferation of keratinocytes, culminating in the formation of the characteristic wart morphology [2].

The clinical manifestation of verruca vulgaris is varied, encompassing small, elevated lesions to large forms that might be very extensive [3]. In rare cases, verruca vulgaris can present as a cutaneous horn, a hyperkeratotic projection that can be mistaken for other skin lesions [4]. The complex interaction between HPV and the host's immune response is significantly influenced by genetic predispositions, particularly polymorphisms in human leukocyte antigen (HLA) genes, which may be crucial in determining susceptibility to and resolution of HPV-induced warts [5]. Treatment modalities for verruca vulgaris are diverse and encompass topical steroid medicines, cryotherapy, laser interventions, and immunotherapy. Cryotherapy is a prevalent technique owing to its efficacy in promoting wart regression via targeted tissue death. The recurrence rate of warts following therapy can be considerable, requiring numerous treatment sessions. Recent studies have explored the use of intralesional bleomycin injections as a novel therapeutic approach, demonstrating promising results in cases resistant to conventional treatments with temporary hyperpigmentation and mild pain. Two intralesional bleomycin injections administered at one-month intervals yielded outstanding results, with no recurrence, ulceration, or scar formation observed [6].

Alternative medicines like homeopathy have been explored alongside conventional treatments, although evidence substantiating their effectiveness is scarce [7]. However infrequent, complications linked to verruca vulgaris may encompass the emergence of squamous cell carcinoma in chronic lesions. This relationship highlights the necessity of effectively monitoring and controlling verruca vulgaris, especially in individuals with widespread or chronic lesions [8].

Traditional therapeutic approaches include various interventions such as the application of topical pharmacological agents, the performance of surgical excision procedures, and the implementation of cryotherapy techniques; however, it is essential to acknowledge that these treatment modalities may exhibit certain inherent limitations and may also lead to a range of undesirable side effects [9,10]. Siddha medicine, recognized as an ancient and time-honored medical tradition, offers a comprehensive and holistic framework for effectively managing and treating verruca vulgaris. The primary objective of this case report is to systematically document and evaluate the therapeutic efficacy of Siddha medicine, Pachaieruvai.

## Case presentation

Background

This case report provides a comprehensive analysis of the clinical manifestations observed in a 60-year-old female patient presenting with hyperkeratotic, verrucous surface and non-painful growth lesions confined to the left malar region, which have endured for six years. The patient identified the significant facial giant verrucae vulgaris based on the evident clinical characteristics. The patient's previous medical and family histories revealed no notable discoveries. She has not received any prior therapy.

Clinical assessment

The dermatological examination identified hyperkeratotic, verrucous surface and non-painful papular lesion (3.2 x 2.6 x .6 cm), in the left malar region.

Siddha intervention

**Figure 1:**
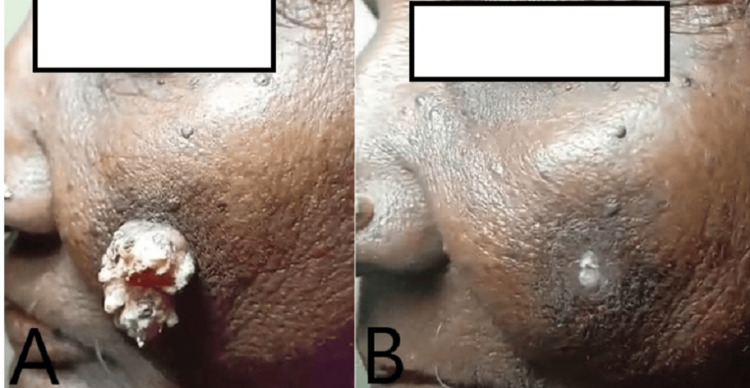
Giant Verruca vulgaris before treatment (A), After treatment (B)

The therapy was performed with the requisite written consent. Pachaieruvai was applied externally for five consecutive days. The progression of the facial skin lesion prior to treatment (A) and seven days post-treatment (B) is illustrated in Figure 1.

Follow-up

In the tri-monthly follow-up assessment following the intervention, no adverse effects or recurrences were observed. Although photos are unavailable due to privacy concerns, we have provided a comprehensive account of the patient's clinical progress, treatment efficacy, and follow-up assessments to ensure the report remains informative. While additional follow-up imaging is crucial for monitoring non-recurrence, obtaining more images was challenging due to the patient’s unwillingness, likely related to concerns about radiation exposure and inconvenience. This limitation has been documented in the manuscript, and we have included all available evidence to support.

## Discussion

This study delineates a preliminary examination regarding the management of giant verruca vulgaris by utilizing Pachaieruvai, a traditional therapy rooted in Siddha medicinal practices, as delineated within Siddha literature. The composition of pachaieruvai encompasses vellaipadanam (arsenic trioxide), aridharam (arsenic trisulphide), thurusu (copper sulfate), karchunnam (calcium carbonate), and kungiliyam (resin of shorea robusta) [11].

Research has established that vellaipaadanam, commonly referred to as arsenic trioxide, which constitutes a crucial element of pachaieruvai, demonstrates both anti-glioma and antiviral properties. It has been documented that it restricts the proliferation of glioma cells while facilitating programmed cell death [12]. Arsenic trioxide (As2O3) profoundly activates apoptotic pathways in HPV16 DNA-immortalized human cervical epithelial cells (HCE16/3 cells) [13]. Aritharam, identified as arsenic trisulphide, possesses cytotoxic characteristics by obstructing the formation and growth of solid tumors [14]. Thurusu, known as copper sulfate, exhibits a cytotoxic effect that impedes tumor proliferation and possesses antiviral activity [15,16]. It induces cytotoxic outcomes by generating reactive oxygen species (ROS) [17]. Karchunnam, recognized as calcium carbonate, displays cytotoxic attributes [18]. Likewise, red kungiliyam, the resin derived from shorea robusta, has been shown to possess cytotoxic effects that inhibit cellular proliferation [19].

Mode of action

The inhibition of cellular proliferation by the constituents of pachaieruvai can be ascribed to mechanisms involving cellular apoptosis and antiviral efficacy. In the discussion segment, I presented a comprehensive elucidation of the implications of these constituents.

Case benefits

The hyperkeratotic papule covering the left malar region was destroyed. During the three-month follow-up visit after the final treatment, no adverse effect or recurrence was observed.

Limitations of the study

Case studies primarily focus on individual patients, creating difficulties in generalising findings to broader populations. The manifestation and severity of Verruca vulgaris can vary considerably among individuals, complicating the extrapolation of findings from a single instance to the broader population affected by the disorder. Although case studies help clarify correlations between causative elements and outcomes, they can not demonstrate definitive cause-and-effect linkages. Without rigorously controlled trials or large observational research, it is difficult to determine if specific factors significantly influence the onset or progression of Verruca vulgaris. The restricted scope of case studies may only sufficiently cover a portion of the spectrum of Verruca vulgaris signs or treatment responses.

Additional research is essential to comprehend the degree of variability linked to the illness and assess the effectiveness of treatment methods. The inclination to publish case studies featuring unusual or remarkable findings is increased, potentially resulting in a bias towards extreme or anomalous cases in the current literature. This tendency may create a misleading depiction of the frequency and normal development of verruca vulgaris. Case studies, despite their limitations, can improve the understanding of therapeutic practices, inspire future research questions, and demonstrate the complexities of managing verruca vulgaris in practical settings; however, it is crucial to interpret these findings with caution and to complement them with data from diverse research methodologies to achieve a comprehensive understanding of the issue.

## Conclusions

This research endeavor chiefly examines the Siddha approach utilized for the therapeutic intervention of verruca vulgaris. The empirical evidence and observations suggest that Siddha pharmacotherapy is effective in the management of verruca vulgaris. Implementing pachaieruvai within this methodology presents many advantages, including economic viability, temporal efficiency, minimal dosage requirements, and a precise localized therapeutic effect. Furthermore, its financial viability positions it as an advantageous alternative within the therapeutic landscape. In cases of verruca vulgaris, free from reappearance associated with conventional Siddha practices, an all-encompassing healing approach that addresses the complete spectrum of a patient's ailments and worries could be advantageous. This case study outlines a practical and cost-effective strategy to address the prevalent issue of facial verrucae vulgaris, thereby enhancing the understanding of the medical implications and socio-economic factors related to their management and treatment. Continued study is crucial to clarify the mechanisms of pachaieruvai against verruca vulgaris and to design more effective treatment strategies.
